# The World Health Organization's Actions Within the United Nations System to Facilitate a Whole-of-Society Response to COVID-19 at Country Level

**DOI:** 10.3389/fpubh.2021.831220

**Published:** 2022-01-18

**Authors:** Gina Samaan, Michelle McPherson, Johan Eidman, Offeibea Obubah, Jean-Pierre Baptiste, Lianne Kuppens, Kai Von Harbou, Miftahul Fahmi Sembiring, Shambhu Acharya, Peter Graaff

**Affiliations:** ^1^Health Emergencies Programme, World Health Organization, Geneva, Switzerland; ^2^Department of Country Strategy and Support, World Health Organization, Geneva, Switzerland; ^3^World Health Organization, Bamako, Mali; ^4^World Health Organization, Tashkent, Uzbekistan; ^5^World Health Organization, Cox's Bazar, Bangladesh

**Keywords:** COVID-19, whole-of-society, World Health Organization, country, United Nations, pandemic, response

## Abstract

The coronavirus disease (COVID-19) pandemic highlighted that managing health emergencies requires more than an effective health response, but that operationalizing a whole-of-society approach is challenging. The World Health Organization (WHO), as the lead agency for health within the United Nations (UN), led the UN response at the global level through the Crisis Management Team, and at the country level through the UN Country Teams (UNCTs) in accordance with its mandate. Three case studies—Mali, Cox's Bazar in Bangladesh, and Uzbekistan—provide examples of how WHO contributed to the whole-of-society response for COVID-19 at the country level. Interviews with WHO staff, supplemented by internal and external published reports, highlighted that the action of WHO comprised technical expertise to ensure an effective whole-of-society response and to minimize social disruption, including those affecting peacekeeping in Mali, livelihood sectors in Cox's Bazar, and the education sector in Uzbekistan. Leveraging local level volunteers from various sectors led to both a stronger public health response and the continuation of other sectoral work. Risk communication and community engagement (RCCE) emerged as a key theme for UN engagement at country level. These collective efforts of operationalizing whole-of-society response at the country level need to continue for the COVID-19 response, but also in preparedness for other health and non-health emergencies. Building resilience for future emergencies requires developing and exercising multi-sectoral preparedness plans and benefits from collective UN support to countries. Coronavirus disease had many impacts outside of health, and therefore emergency preparedness needs to occur outside of health too.

## Introduction

Managing the health risks and reducing the socioeconomic impact of emergencies requires a whole-of-society approach (1), defined as a governing arrangement where public agencies directly engage non-state stakeholders in a collective decision-making process that is consensus-oriented, deliberative, and aims to make or implement public policy or manage public programs or assets (2). The World Health Organization (WHO) promotes the full and effective contributions of all relevant stakeholders to the risk management of emergencies, with stakeholders including individuals, families and communities, governments, intergovernmental organizations, the private sector and industry, faith groups, civil society, the media, academia, research bodies, and voluntary associations (1). Having a whole-of-society approach has been highlighted as a key component of successful coronavirus disease (COVID-19) responses in the few reviews publicly available (3–8).

Early in the pandemic, WHO recognized that the impact of COVID-19 would expand beyond the health sector requiring a coordinated whole-of-society approach. In February 2020, the WHO Director-General requested activation of the United Nations (UN) Crisis Management Policy by the UN Secretary-General—the highest possible level of crisis alert in the UN system (9), and the first activation for a public health event. World Health Organization, as the directing and coordinating authority on international health within the UN system, chaired the COVID-19 Crisis Management Team, to coordinate the UN strategies, policy decisions, and plans; and joint UN action to scale up country-level operations for public health, human rights concerns, broader socioeconomic-related issues, and travel and trade (9).

Three critical components of the UN response to COVID-19 were the Strategic Preparedness and Response Plan for 2019-nCoV (SPRP) (10), the COVID-19 Global Humanitarian Response Plan (GHRP) (11), and the UN framework for the immediate socio-economic response to COVID-19 (SERF) (12). These response frameworks leveraged the expertise and delivery capacities of UN entities to build a whole-of-society approach for country preparedness and response to COVID-19 by harnessing the resources of public and private sectors and civil society (9). The 131 UN Country Teams (UNCTs) that serve 162 countries and territories (12), led by UN Resident Coordinators, were the coordination forum for the UN system and the three plans at the country level.

Multi-sectorial collaboration during health emergencies has been recognized as an essential, and yet challenging, component of the response. The independent Review Committee on the Functioning of the International Health Regulations, in their review of the COVID-19 response, highlighted the need for multisectoral collaboration, and for WHO to work with Member States to engage stakeholders beyond the health sector to identify and address country level gaps in preparedness (13). The Global Preparedness Monitoring Board in their 2020 annual report confirmed that the impact of pandemics goes far beyond their immediate health effects, and that the COVID-19 pandemic has demonstrated the importance of protecting lives and livelihoods, and widening preparedness to make education, social, and economic sectors “pandemic proof” (14). Although acknowledged as a critical component of emergency response, there is little evidence of how to operationalize a whole-of-society response.

World Health Organization, as the lead agency for health within the UN, and in accordance with its mandate (15), provided leadership, policy dialogue, strategic support, technical assistance, and service delivery at the country level for the COVID-19 response. Through three case studies, this paper demonstrates that WHO contributed to the whole-of-society response to COVID-19 at the country level through the UN system.

The three case studies were selected according to the following criteria: (a) different contexts including low- and middle-income countries and fragile settings with vulnerable populations, (b) different geographic regions, (c) large UN presence of 15 or more agencies, (d) agreement by the WHO Representative for interview. A convenience sample were selected from the collation of 70 case studies reported on in 2020 focusing on WHO's country level work in response to COVID-19 (16) to provide a qualitative account while respecting manuscript length and journal style. The three case studies focused on Mali, Cox's Bazar in Bangladesh, and Uzbekistan.

Mali is a low-income country with a long-term UN peace-keeper presence. Cox's Bazar in Bangladesh comprises a large vulnerable population of refugees in a low-to-middle-income country with an international humanitarian response. Uzbekistan is a middle-income country with a UN interagency presence where the UN is a development partner providing technical assistance. The WHO Representative or staff from each was interviewed about the role of WHO within the UN system in the whole-of-society response for COVID-19. The information gathered from the interviews is supplemented by internal and published reports.

## Case Study 1: Mali

Mali is a fragile state in Africa with a large UN presence, consisting of 21 UN entities plus the Multidimensional Integrated Stabilization Mission in Mali (MINUSMA), established by the UN Security Council in 2013. Collectively, the UN in Mali comprises almost 19,000 staff and personnel, including 13,000 military, 5,600 civilians, and 1,560 employees of agencies (16). After decades of instability, the 2015 peace accord is still being implemented, and there is continued insecurity in central and northern Mali.

Prior to the COVID-19 pandemic, although there were many joint UN projects, most UN agencies operated within their own functions and mission. This changed for the COVID-19 response—as the full machinery of the UN operated “as one” in an unprecedented way (16). The National Crisis Management Team comprised the UNCT, humanitarian organizations, and MINUSMA and was led by the UN Resident Coordinator; seven working groups were established to analyse, monitor, and ensure coordination in logistics and procurement, financing, information systems, human resources, and security (9). World Health Organization, as the technical lead of the UN team, provided evidence, guidance, and standards for decision making and the work of the multisectoral UN team. World Health Organization provided weekly COVID-19 epidemiological updates to the National Crisis Management Team, the UN Mission, and partners.

World Health Organization provided the link between the government and the UN Resident Coordinator for the COVID-19 response, based on their well-established relationship with the Ministry of Health and Social Development (MHSD). World Health Organization mobilized staff from other UN agencies to provide technical support to the government across whole-of-society activities. Of the 25 UN team members, seven were from non-WHO UN agencies including from UN Children's Fund (UNICEF) for risk communication and community engagement (RCCE), the UN Development Programme (UNDP) for program management and the World Food Program (WFP) for logistics and supply chain management. This structure meant that for the first time the National Health Authority collaborated with multiple UN agencies.

The enabling role of WHO is illustrated by the public health and social measures adopted by MINUSMA to overcome the challenges in operating within COVID-19 restrictions. Guided by WHO recommendations and working closely with national authorities, MINUSMA implemented quarantine, body temperature checks before accessing camps, social distancing, mask wearing, and COVID-19 pre-deployment training to ensure its continued operation (17). Multidimensional Integrated Stabilization Mission in Mali, with technical guidance from WHO, also supported the national COVID-19 response through the provision of relevant equipment, material, and infrastructure; more than 50 tons of protective materials were dispatched by September 2020 (18). Another example of WHO's enabling role was technical guidance for COVID-19 protocols in schools and the provision of information materials to the education sector to ensure their continual operation alongside efforts from UNICEF and other agencies (18). The UN's collective efforts were also demonstrated in the legislative elections held in Mali on 29 March and 19 April 2020, where the UN supported government authorities including the Ministry of Territorial Administration and Decentralization and MHSD to operationalize public health measures including the provision of infection prevention and control commodities at polling stations.

Getting appropriate RCCE messaging to hard-to-reach populations was a challenge in Mali that was overcome by a whole-of-society approach supported by WHO (19). The UN peace radio station, Radio Mikado, relied on WHO information for its crucial role in risk communication, with the WHO Representative a guest speaker (20). World Health Organization guidance was also critical for the innovative use of speaker drones to deliver RCCE messages to remote areas. Drones were used to distribute RCCE materials developed by WHO, in conjunction with the UN and the MHSD. Messages were translated into the appropriate languages, loaded onto the drones, and sent to relevant villages for announcement (21). A feedback loop was established through the village health cadre who noted the utility of drones in real-time authoritative information provision. As this mechanism was successful for the delivery of RCCE messages, it was also used for the vaccine roll-out to provide messages to locations before the vaccine arrived. This modality allowed RCCE messages to reach previously hard-to-reach populations, and those without access to television, radio, and internet.

## Case Study 2: Cox's Bazar

Cox's Bazar, in Bangladesh, comprises a large refugee population spread across 34 highly congested subcamps along the Bangladesh/Myanmar border. The international response coordination in Cox's Bazar is managed under a situation specific mechanism led by a Strategic Executive Group at the national level and through the Inter Sector Coordination Group (ISCG) in Cox's Bazar. The ISCG comprised ten sectors and six inter sector working groups that provide humanitarian assistance to close to 900,000 Rohingya refugees (22). The WHO Emergency Sub-office in Cox's Bazar, in collaboration the district level health administration, chairs the Health Sector of the ISCG.

Guided by the WHO Emergency Response Framework (23), WHO Bangladesh established an incident management system soon after the largest influx of refugees from Myanmar in 2017 that effectively managed many outbreaks and natural disasters prior to COVID-19 (24, 25). Both the WHO incident management system and the ISCG were leveraged for COVID-19, highlighting the benefit of having existing mechanisms using a whole-of society response for emergencies (26). The COVID-19 response was also extended to the entire population of Cox's Bazar district.

Prior to the first COVID-19 cases in the district, a COVID-19 response plan was developed that outlined key activities across 11 identified thematic pillars of the national COVID-19 response plan (27). A dedicated COVID-19 related Crisis Management Team for Cox's Bazar was established, initially comprised of the UN Resident Coordinator and Heads of Agencies of WHO, International Organization for Migration (IOM), UN High Commissioner for Refugees (UNHCR), UNICEF, and WFP as well as the Head of Sub-office of WHO and the senior coordinator of ISCG. This forum enabled frequent and direct interagency decision making and was a key facilitator of operational decision-making between the field and national decision makers.

The response to COVID-19 in Cox's Bazar provides many examples of how WHO supported whole-of-society COVID-19 activities. World Health Organization provided technical updates to the Heads of UN agencies and the ISCG, and between UN agencies, local community groups, donors/partners, and embassies to facilitate decision making, allocate resources, and mobilize funds. In collaboration with the technical working groups, the health sector developed an orientation package and online awareness training sessions for healthcare workers to ensure mainstreaming of cross-cutting themes including gender, protection from sexual exploitation and abuse, gender-based violence, protection, and child protection in the response. Prior to the reopening of the destination for domestic tourism, in collaboration with local government, WHO provided technical advice, training, and information materials on operating safely within the COVID-19 context to various sectors, including the tourism, agriculture, and livelihood sectors.

One unique example of the role of WHO in the whole-of-society response to COVID-19 in Cox's Bazar was the provision of technical advice and coordination for repurposing Rohingya volunteers involved in garment manufacturing and tailoring as livelihoods activities to produce cloth face masks (27). World Health Organization's role in this initiative was to provide the specifications for the fabric masks (28), the quality standards for local production, and in collaboration with the livelihood sector partners including UNHCR, IOM, and WFP, the government counterparts including the district's Civil Surgeon and regulatory authority, support the system for UN agencies that normally work with the production sector. More than one million masks were made and distributed in the camps and nearby community. This initiative contributed to the health response and improved the refugees' micro economy (29).

Restriction to the camps was another challenge for the whole-of-society response as many volunteers from non-health sectors such as protection, education, and nutrition were unable to continue their work. To overcome this challenge, WHO utilized these volunteer networks in partnership with the other UN agencies for health activities, which also enabled their usual volunteer roles. The volunteers contributed to the community surveillance system established prior to COVID-19 (30), referred cases for testing and treatment, assisted with vaccination programs, and provided the backbone of RCCE. With partners, WHO provided training for 3,600 humanitarian workers and over 2,000 volunteers involved in the COVID-19 response on how to protect themselves from COVID-19 while working. By June 2020, every household was visited once a week by these community volunteers.

The training provided to the volunteer networks enabled RCCE efforts as the volunteers disseminated these messages whilst completing their usual work (31). This intersectoral involvement in RCCE successfully alleviated the infodemic within the camp, as evidenced by improved outpatient attendance and health seeking behavior of the population (32). The RCCE feedback loop was also supported by WHO, as it enabled structured feedback to the government, UN agencies, and non-government partners at national and local level to improve the response (33). In collaboration with partners, messages were also delivered by WHO staff through weekly audio podcast program on local radios (34).

## Case Study 3: Uzbekistan

Uzbekistan is a middle-income country in central Asia that has a UN interagency presence with a UNCT that comprises 24 UN agencies (35). World Health Organization works closely with the Ministry of Health (MOH), and prior to COVID-19, established Health Development Partnership meetings to facilitate communication between the UNCT, non-UN development partners, and the government.

A UN Crisis Management Team for COVID-19 was established and was co-chaired by WHO. The Crisis Management Team operated according to the national SPRP and the UN framework for the socio-economic response to COVID-19 in Uzbekistan. World Health Organization led the SPRP, contributed to the combined UN/WHO sitreps, was a co-chair of most COVID-19 related meetings, and a vital part of the UN decision making process. This further increase the visibility and added value of WHO within the UN system in Uzbekistan.

There were six COVID-19 taskforces led by the UN and the government: capacity building, procurement, human rights and vulnerable groups, economic and social impact, education, and risk communication. World Health Organization led the capacity building taskforce and had a presence in all others. By MOH request, WHO was the liaison between the MOH and other UN agencies and the conduit between the COVID-19 taskforces. The Health Development Partnership meetings continued as the intersectoral communication and collaboration mechanism between the UN, non-UN partners, and government. World Health Organization presented technical information to each taskforce as needed and reviewed taskforce documents prior to distribution to the government—a “translator.” The COVID-19 response cemented that intersectoral collaboration is crucial for advancing the health agenda with WHO as a key contributor.

One example of WHO working across the whole-of-society response in Uzbekistan was in the education taskforce. Working closely with UNICEF and the UN Educational, Scientific, and Cultural Organization (UNESCO), WHO contributed to technical guidance on school closures, required school infrastructure, such as building ventilation and the safe reopening of schools. Information, education, and communication materials were developed in collaboration with the MOH, Ministry of Education, and Ministry of Pre-School Education with more than 6 million children in schools, 1.4 million children in pre-schools, 2 million parents received information, and 14,000 preschool institutions receiving materials (35).

Online training mechanisms developed by WHO, in conjunction with the MOH Post Graduate Medical Institute, allowed experts to train large groups of healthcare workers in the public and private sector from across Uzbekistan (500 people per session) on COVID-19 related issues and topics, with the training materials made available to partners outside of the health sector.

## Discussion

The COVID-19 pandemic has highlighted that responding to a global infectious disease pandemic has far-reaching implications outside the health sector that requires a whole-of-society approach. The UN, with its existing global presence, and guided by the three overarching global plans for health, humanitarian, and socio-economic response (10–12), was well-placed to support the whole-of-society response to the COVID-19 pandemic at the country level. The three case studies presented demonstrate that WHO contributed to the whole-of-society response at the country level, in addition to leading the health response as per its mandate. World Health Organization provided leadership, technical, and policy guidance and was the facilitator between government and the UN, which contributed to the whole-of-society response. Risk communication and community engagement was another integral component of the response that benefitted from this collaborative governance approach observed within the three contexts. Whilst the focus of the paper is on the actions of WHO, the authors acknowledge that the whole-of-society response at country level was a collaborative effort between all UN agencies, governments, and partners. The collective efforts supported positive outcomes including sustained community engagement, continued education, and livelihood initiatives while maintaining a health first approach to minimize COVID-19 risks.

The COVID-19 pandemic has impacted everyone globally, in every facet of their lives. There has been a drastic decrease in the human development index globally (36), with many measures implemented to stop transmission of COVID-19 disrupting society, reducing essential health services, and negatively affecting livelihoods. Schoolchildren worldwide have lost more than 1.8 trillion hours of in-person learning due to COVID-19 lockdowns (37), the 2021 global tuberculosis report shows a global decrease in newly diagnosed cases, access to treatment, and global spending on TB, as well as most global TB targets being off track (38). At the country level, WHO, through the UN system, provided technical information and policy guidance to minimize this social disruption. Technical guidance provided to MINUSMA in Mali ensured its continual operation throughout the pandemic, and advice provided to the tourism, agriculture, and livelihood sectors in Cox's Bazar, and the education sector across all three locations, enabled reopening within the COVID-19 context. Overcoming the challenges to continue the whole-of-society activities within refugee camps by leveraging volunteers to the health response and contributing to livelihood projects such as mask-making, provide further examples of how WHO, using their existing mechanisms, partnerships, and networks, provided technical expertise to ensure an effective whole-of-society response.

World Health Organization also provided leadership at the country level within the UN response globally, with 87% of WHO country offices reporting that they led the COVID-19 response within the UNCTs and 94% also reporting that their role within the UNCT expanded due to the pandemic (39). The three case studies elaborate this aspect of WHO's role: WHO was the technical lead of the UN team in Mali, a key member of the existing interagency decision-making body that formed the Crisis Management Team in Cox's Bazar, and co-lead with the UN Resident Coordinator of the Crisis Management Team in Uzbekistan. Having country level Crisis Management Teams with multi-agency representation, underpinned by the three global plans—SPRP, GHRP, and SERF—provided a shared imperative that strongly accelerated progress on joint working within the UN. All three cases studies described how the UNCTs worked together, with WHO often providing the link between the UN and local government, based on their existing relationships with the health sector, but expanded to all sectors for the COVID-19 response.

Risk communication and community engagement has been highlighted as an integral component of the COVID-19 response that has been exacerbated by an “Infodemic” including misinformation and rumors (40). The three case studies show that WHO contributed to RCCE efforts at the local level, by providing technical information distributed though information, education, and communication materials, WHO shared validated information, trained intersectoral volunteers with correct messaging, and supported innovation including the drone message delivery system in Mali.

The COVID-19 pandemic has emphasized that managing health emergencies requires more than an effective health response, but that operationalizing a whole-of-society approach is challenging. Providing the actions and inputs from WHO only in this perspective article is a limitation which can be strengthened by further work to assess and present the perspectives of other UN agencies and beneficiaries in whole-of-society response. So that these experiences are not lost, WHO should continue the whole-of-society interactions within the COVID-19 response through the UN system, and in preparedness for other health and non-health emergencies. There are opportunities to sustain these efforts beyond the COVID-19 pandemic, such as cross-purposing volunteers within different sectors, optimizing use of digital technologies for RCCE, and strengthening platforms for online education to minimize the negative impact of future crises on children. Building resilience for future emergencies can also be achieved more broadly through developing and exercising multi-sectoral preparedness plans and through collective UN support to countries in prevention, risk governance, and forging critical partnerships. As stated in the SPRP, no single agency or organization can prepare for or respond to such an event on its own (10).

## Data Availability Statement

The original contributions presented in the study are included in the article/supplementary material, further inquiries can be directed to the corresponding author/s.

## Author Contributions

GS, MM, JE, OO, SA, and PG: conceptualization. GS, MM, JE, OO, J-PB, LK, KV, and MFS: data collection and interpretation. GS, MM, JE, OO, and KV: drafting the manuscript. GS, MM, JE, OO, J-PB, LK, KV, MFS, SA, and PG: review and revising manuscript. All authors contributed to the article and approved the submitted version.

## Conflict of Interest

The authors declare that the research was conducted in the absence of any commercial or financial relationships that could be construed as a potential conflict of interest.

## Publisher's Note

All claims expressed in this article are solely those of the authors and do not necessarily represent those of their affiliated organizations, or those of the publisher, the editors and the reviewers. Any product that may be evaluated in this article, or claim that may be made by its manufacturer, is not guaranteed or endorsed by the publisher.
